# Wave Propagation of Porous Nanoshells

**DOI:** 10.3390/nano9010022

**Published:** 2018-12-24

**Authors:** Behrouz Karami, Davood Shahsavari, Maziar Janghorban, Rossana Dimitri, Francesco Tornabene

**Affiliations:** 1 Department of Mechanical Engineering, Marvdasht Branch, Islamic Azad University, Marvdasht 73711-13119, Iran; shahsavari.davood@miau.ac.ir (D.S.); maziar.janghorban@miau.ac.ir (M.J.); 2 Department of Innovation Engineering, Università del Salento, Lecce 73100, Italy; rossana.dimitri@unisalento.it

**Keywords:** doubly-curved nanoshell, generalized non-local strain gradient theory, higher-order shear deformation shell theory, porous materials, wave propagation

## Abstract

This study aims at investigating the wave propagation of porous nanoshells. The Bi-Helmholtz non-local strain gradient theory is employed in conjunction with a higher-order shear deformation shell theory, in order to include the size-dependent effects. The nanoshells are made of a porous functionally graded material (P-FGM), whose properties vary continuously along the thickness direction. A variational approach is here applied to handle the governing equations of the problem, which are solved analytically to compute the wave frequencies and phase velocities as function of the wave numbers. The sensitivity of the wave response is analyzed for a varying porosity volume fraction, material properties, non-local parameters, strain gradient length scales, temperature, humidity, and wave numbers. Based on the results, it is verified that the size-dependence of the response is almost the same to the one of plates, beams and tubes.

## 1. Introduction

Over the past few years, the research on nanomaterials has gained an increasing attention in the scientific community. Due to the benefits of nanomaterials, many engineering components are gradually becoming lighter, smaller, stronger, and less expensive. Recent requirements in design and manufacturing has led to an increased development of nanoshells, carbon nanotubes, and paramagnetic nanoparticles for many engineering applications, e.g., biomedicine [1], drug or gene delivery [2], aerospace facilities [3] automobile industry [4] and energy devices [5]. For example, gold-shelled nanoparticles, which are spherical nanoparticles, can be used in cancer therapy as well as in bio-imaging enhancement [6]. The capability to control both wavelength-dependent scattering and absorption of nanoshells provides a great opportunity to design nanoshells, offering diagnostic and therapeutic abilities in single nanoparticles [7].

Shell structures are typically curved lightweight three-dimensional solids whose thickness is smaller than the other two dimensions, whereby a large number of shell theories have been proposed and studied to this aim [8,9,10,11,12,13,14,15,16,17,18,19,20,21,22,23,24,25]. An Equivalent Single Layer (ESL) theory [8,9], for example, represents a sensible way of analyzing the behavior of shell structures, whereas other approaches, such as the 3D-elasticity theory or the Layer-Wise (LW) theories, could be generally more expensive. Thus, the ELS allows us to define Higher-order Shear Deformation Theories (HSDTs), as commonly adopted to study the mechanical behavior of beams, plates and shells [22,23,24,25,26]. HSDTs satisfy the shear deformation effects on the bottom and upper surfaces of the shell structures without considering any shear correction factor, if compared to the First-Order Shear Deformation Theories (FSDTs). Moreover, using the HSDTs, a lower number of variables is involved in the equilibrium equations of the problem. On the other hand, beams, plates and shells made of advanced or biological materials can feature very large deformations, associated with a large thickness stretching, as typically occurs in functionally graded materials (FGMs) and arteries under an internal pressure [27,28]. Carrera et al. [29] showed that classical continuum theories are disabled to consider the thickness stretching effect due to the main assumption of constant transverse displacement through the thickness, and studied this effect on the mechanical behavior of FGM plates by means of finite element approximations. Thus, a great attention in the scientific community has been devoted to investigate the mechanical behavior of FGM structures at the micro/nano-scale, when the thickness stretching effect is included as assumption [29,30,31,32,33,34,35,36,37,38,39,40]. Shahsavari et al. [41] developed quasi-3D theories for the study of the free vibration response of porous functionally graded materials (P-FGMs), while considering both shear deformation and thickness stretching. Hence, this study proposes a HSDT including the stretching effect in FGM shells.

The wave propagation represents a multi-physics problem, including geophysics, acoustics, blood flow, non-destructive evaluation, hydrodynamics, and other applications in which waves travel. The main distinction between transverse and longitudinal waves, considers both directions of oscillation and propagation. For an accurate analysis of structures, wave propagation is one of the key questions for researchers who operate in the ultrasonic inspection techniques and structural health monitoring [42]. Due to the non-linear behavior of the shell structures under high-level loading conditions, an adequate information on waves propagation for varying material properties and geometries represents a key aspect for the numerical models of such structures [43]. To date, a large amount of works has been carried out on the wave propagation analysis of shells [13,14,15,16,17,19,44]. A pioneer study on the topic was presented by Fuller and Fahy [13], who studied the wave characterization of cylindrical elastic shells filled with a fluid. In 1996, Mead [14] presented a brief review paper on the wave propagation in continuous periodic structures, namely, beams, plates or cylindrical shells. Yuan and Hsieh [15] proposed a 3D solution for the free harmonic wave propagation problem in composite laminated cylindrical shells. The propagation of waves in thin shells was also examined by Solaroli et al. [16], by using an eigenvalue solution. A different elasto-dynamic solution was proposed by Wang et al. [17] for the study of the stress wave propagation of thick laminated spherical shells with arbitrary thickness. Moreover, Dai and Wang [45] applied an analytical method to study the wave propagation of laminated piezoelectric spherical shells subjected to thermal shocks and electric excitations, and they verified the sensitivity of the wave propagation phenomenon to the external loading. Further detail about the introduction and modeling of the displacement field, the strain components and solution methods for the study of the wave motion within elastic solids, can be found in the book by Graff [19], including stings, thin rods, membranes, plates and shells. 

As far as FGM is concerned, this represents a common class of composite materials widely used in many engineering fields, namely, aerospace, biomechanics, optics and nuclear energy. This material is made of two or more segments whose distribution varies randomly within the volume. However, during a fabrication process of these composite materials, porosities and voids may develop owing to the difference in solidification temperature of material segments [46]: this could threaten the global safety factor of constructions. This means that the analytical and numerical studies on FGMs cannot disregard the effect of porosity. Moreover, it is well known that the variation of porosity within the thickness yields a considerable variation in the mechanical properties [42,47,48]. On the other hand, the experimental evaluation of the porosity effect at a micro/nanoscale represents a difficult and expensive task. This has increased the scientific interest in the investigation of the porosity effect at a micro/nanoscale, usually approached through the size-dependent continuum theories. To cite some papers about this issue, Shahsavari et al. [49] applied a size-dependent quasi-3D theory for the study of the shear buckling response of a P-FGM nanoplate in a hygrothermal environment. The authors demonstrated the influence of the environmental condition and porosity distribution on the shear buckling response of nanoplates. Moreover, a quasi-3D size dependent including thickness stretching effect for wave propagation analysis of FGM nanoplates under a hygrothermal environment was suggested by Karami et al. [50]. As also detailed in the review paper [25], the size-dependent 3D theories are able to capture the size effect at micro/ nanoscale in an easy manner, with a reduced computational cost. 

A further comparative evaluation between size-dependent theories with respect to the experimental data available in literature, and predictions based on size-independent classical continuum theories, can be found in [51,52,53,54]. In some recent works, [55,56,57], the non-local elasticity theory (NET) has been combined to the strain gradient theory (SGT), to yield the non-local strain gradient theory (NSGT). Different applications of the NSGT can be found in Refs. [58,59,60,61,62,63,64,65,66], where the mechanical variation of materials and structures is discussed for a varying non-local parameter (e.g., the softening-stiffness mechanism) or strain gradient parameter. Further studies identify some useful small-scale parameters, both experimentally and numerically, according to the molecular dynamics approach (see e.g., [67,68,69,70]). Karami et al. [50], also, suggested a quasi-3D size-dependent approach for the wave propagation analysis of FGM nanoplates in a hygrothermal environment, including the thickness stretching effect. In line with their previous work, Karami et al. [71] investigated the wave propagation response of nanoplates resting on an elastic foundation, under an in-plane magnetic field, and made of temperature-dependent P-FGMs. 

In the recent works [72,73,74], it has been shown that the addition of an extra non-local parameter within a NSGT formulation, gives more accurate results when studying the response of nanostructures. A Bi-Helmholtz non-local strain gradient elasticity theory (B-H-NSGT) was proposed in Refs. [72,73,74] for the study of nanobeams, nanotubes and nanoplates, without any relevant application to nanoshells. Therefore, a possible further development of this formulation could consider the wave propagation problem of P-FGM nanoshells, including some small-scale parameters. A key point of the problem, indeed, is whether the geometry and external conditions can change the size-dependent response of porous structures or not. Some recent works on the topic, have managed the size-dependent wave propagation problem of porous nanobeams [72], nanoplates [71] and nanotubes [75] without considering the possible presence of porosities. Moreover, there is a general lack of work in the literature that illustrates the role of the thickness stretching in wave propagation problems of FGM shells. This kind of problem is here tackled for P-FGM nanoshells in hygrothermal environment, by applying both the B-H NSGT and HSDT with stretching effects. A similar model is demonstrated to be very accurate at different scales, due to the basic assumptions in the governing equations. An analytical solution is considered to check for the wave frequency and phase velocity of the nanostructure. Therefore, this article can represent a pioneer reference for an accurate analysis of nanoshell structures by combining the HSDT with stretching effects and the B-H NSGT. Hence, the present model may be selected and developed for complex non-linear analyses of such structures. The article is organized as follows. Section 2 describes the material properties distribution of non-porous materials and the governing equations of the problem according to the B-H NSGT and HSDT. Section 3 gives some additional information about the solution method here proposed. Section 4 illustrates the numerical results obtained according the proposed approach, and some remarkable conclusions are finally summarized in Section 5.

## 2. Theory and Formulation

**Figure 1:**
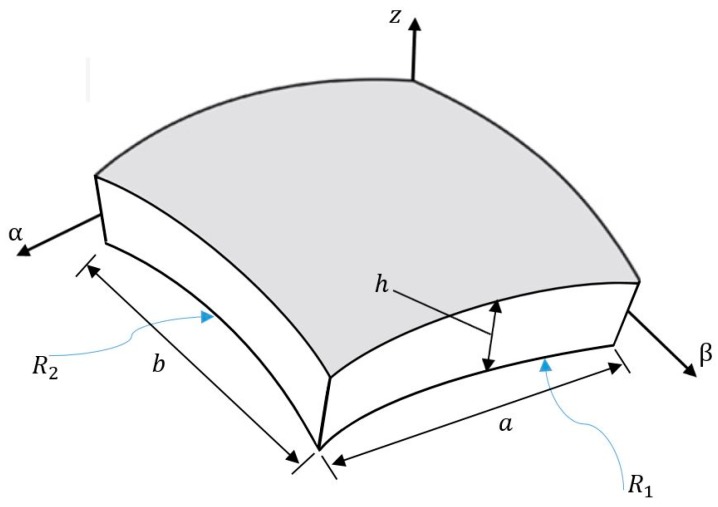
Geometry of a P-FGM doubly-curved nanoshell.

Consider a doubly-curved nanoshell of radius of curvature R, length a, width b and thickness h. The nanostructure is made of P-FGMs and is exposed to a hygrothermal environment (see Figure 1). The bottom surface of the nanoshell is made of a pure metal and the top is made of a pure ceramic.

### 2.1. Porosity-Dependent Functionally Graded Materials

**Figure 2:**
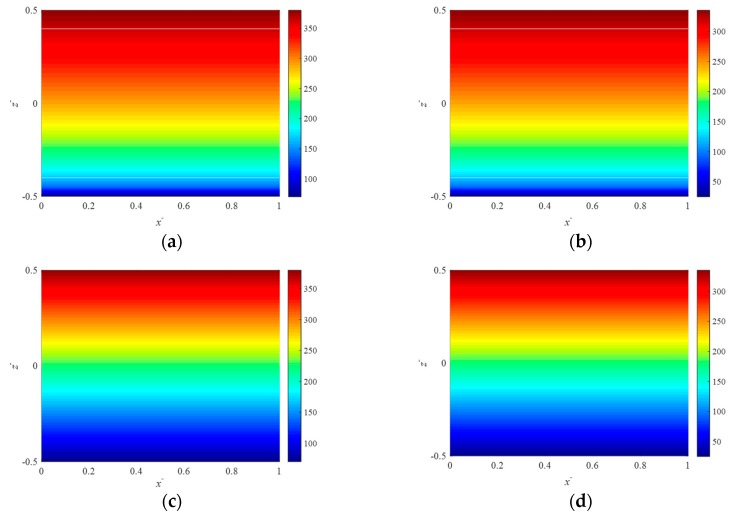
Distribution of the Young’s modulus for *E_c_* = 380 GPa, *E_m_* = 70 GPa at plane $\bar{y}=0.5$, (**a**) *n* = 0.5, *ξ* = 0.0; (**b**) *n* = 0.5, *ξ* = 0.2; (**c**) *n* = 1.0, *ξ* = 0.0; (**d**) *n* = 1.0, *ξ* = 0.2.

A modified power law role is here presented to consider the presence of possible voids (porosities) introduced during the manufacturing process of FGMs. The modified role estimates the effective material properties of FGMs in the following form [46,72,76,77]:

(1) $$P(z)=P_{m}(V_{m}-\frac{\xi }{2})+P_{c}(V_{c}-\frac{\xi }{2})$$

where the porosity volume fraction is always ξ=1, P(z) denotes the effective material properties, the pedexes c and m refer to the ceramic and metal phases of FGMs, respectively; $V_{c}$ and $V_{m}$ are the volume fractions of the ceramic and metal, respectively, with $V_{c}+V_{m}=1$. The volume fraction of the ceramic phase can be estimated as follows:

(2) $$V_{c}={\left(\frac{z}{h}+0.5\right)}^{n}$$

n being the selected power-law index and *z* is the coordinate through the thickness. Hence, the effective material properties of FGMs with porosities can be obtained generally as [42]:

(3) $$P(z)=(P_{c}-P_{m}){\left(z/h+0.5\right)}^{n}+P_{m}-\xi /2(P_{c}+P_{m})$$

where *P*(*z*) can refer to the Young’s modulus E(z), the Poisson’s ratio υ(z), the coefficients of thermal expansion α(z) and moisture expansion β(z). Based on the modified power-law distribution of the effective material properties given by Equations (1)–(3), we plot in Figure 2 the variation of the elastic modulus through the thickness for FGMs.

### 2.2. The General Non Local-Strain Gradient Elasticity Theory

A valid non-local strain gradient model was proposed by Askes and Aifantis [78], based on two small scale parameters. Then, Lim et al. [79] presented a high-order non-local strain gradient model, known as B-H NSGT, including three small scale parameters within the formulation. Thus, the stress-strain relations are expressed as follows:

(4) $$\sigma_{ij}=\sigma_{ij}^{\left(0\right)}-\nabla \sigma_{ij}^{\left(1\right)}$$

$\sigma_{{}_{ij}}^{\left(0\right)}$ and $\sigma_{{}_{ij}}^{\left(1\right)}$ being the classical stress and higher-order stress, respectively, described as follows

(5) $$\sigma_{ij}^{\left(0\right)}=\int_{0}^{L}C_{ijkl}\alpha_{0}\left(x,x^{\prime },e_{0}a\right){\varepsilon^{\prime }}_{kl}\left(x^{\prime }\right)dx^{\prime }$$

(6) $$\sigma_{ij}^{\left(1\right)}=\lambda^{2}\int_{0}^{L}C_{ijkl}\alpha_{1}\left(x,x^{\prime },e_{1}a\right)\nabla {\varepsilon^{\prime }}_{kl}\left(x^{\prime }\right)dx^{\prime }$$

In Equations (5) and (6), $\alpha_{0}\left(x,x^{\prime },e_{0}a\right)$ and $\alpha_{1}\left(x,x^{\prime },e_{0}a\right)$ are the non-local kernel functions, and λ is a material characteristic parameter. The linear non-local differential operator L_*i*_ is applied to both sides of Equation (1), and takes the following form:

𝕃_*i*_ = 1 − (*e*_*i*_*a*)^2^∇^2^  for *i* = 0,1 (7)

$\nabla^{2}$ being the Laplacian operator. It is well known from the literature that differential equations can be solved in a easier manner compared to the integral ones, whereby Lim et al. [79] proposed an extended constitutive equation to threat the higher-order non-local strain gradient theory as follows:

(8) $$\left[1-\mu_{1}^{2}\nabla^{2}\right]\left[1-\mu_{0}^{2}\nabla^{2}\right]\sigma_{ij}=Q_{ijkl}\left[1-\mu_{1}^{2}\nabla^{2}\right]\varepsilon_{kl}-Q_{ijkl}\lambda^{2}\left[1-\mu_{0}^{2}\nabla^{2}\right]\nabla^{2}\varepsilon_{kl}$$

where,

(9) $$\mu_{0}=e_{0}a,\;\mu_{1}=e_{1}a$$

Equation (5) can be redefined in an equivalent form as follows:

(10) $${ℒ}_{\mu }\sigma_{ij}={ℒ}_{l}Q_{ijkl}\varepsilon_{kl}$$

where the linear operators read:

(11) $$\begin{array}{l} {ℒ}_{\mu }=(1-\mu_{1}\nabla^{2})(1-\mu_{0}\nabla^{2})\\ {ℒ}_{l}=(1-\mu_{1}\nabla^{2})-\lambda^{2}(1-\mu_{0}\nabla^{2})\nabla^{2} \end{array}$$

### 2.3. Kinematic Relations

In this section, we describe briefly the HSDT here applied, including stretching effect, to model the doubly-curved shells made of P-FGMs. The displacement field of the present theory can be expressed by [80]:

(12) $$\begin{array}{l} u_{1}=u_{0}\left(\alpha ,\beta ,t\right)+z\phi_{\alpha }-\frac{z^{2}}{2}\frac{\partial w_{1}}{\partial \alpha }-\frac{4z^{3}}{3h^{2}}\left(\frac{\partial w_{0}}{\partial \alpha }+\frac{h^{2}}{4}\frac{\partial w_{2}}{\partial \alpha }+\phi_{\alpha }\right)\\ u_{2}=v_{0}\left(\alpha ,\beta ,t\right)+z\phi_{\beta }-\frac{z^{2}}{2}\frac{\partial w_{1}}{\partial \beta }-\frac{4z^{3}}{3h^{2}}\left(\frac{\partial w_{0}}{\partial \beta }+\frac{h^{2}}{4}\frac{\partial w_{2}}{\partial \beta }+\phi_{\beta }\right)\\ u_{3}=w_{0}\left(\alpha ,\beta ,t\right)+zw_{1}\left(\alpha ,\beta ,t\right)-z^{2}w_{2}\left(\alpha ,\beta ,t\right) \end{array}$$

where $u_{i}$ (*i* = 1–3) denote the displacements components within the shell domain; $u_{0}$, $v_{0}$, and $w_{0}$ are displacement components of the mid-plane along the shell axis; $\phi_{\alpha }$ and $\phi_{\beta }$ are the rotations of the transverse normal at z=0; $w_{1}$ and $w_{2}$ are the parameters related to the thickness stretching per unit of thickness; t refers to the time [27]. The non-zero strain-displacement relations can be expressed as:

(13) $$\begin{array}{l} \varepsilon_{\alpha \alpha }=\frac{\partial u_{1}}{\partial \alpha }+u_{2}+\frac{u_{3}}{R_{1}};\varepsilon_{\beta \beta }=\frac{\partial u_{2}}{\partial \beta }+u_{1}+\frac{u_{3}}{R_{2}};\varepsilon_{zz}=\frac{\partial u_{3}}{\partial z};\\ \gamma_{\alpha z}=\frac{\partial u_{1}}{\partial z}+\frac{\partial u_{3}}{\partial \alpha }-\frac{u_{1}}{R_{1}};\gamma_{\beta z}=\frac{\partial u_{2}}{\partial z}+\frac{\partial u_{3}}{\partial \beta }-\frac{u_{2}}{R_{2}};\gamma_{\alpha \beta }=\frac{\partial u_{1}}{\partial \beta }+\frac{\partial u_{2}}{\partial \alpha } \end{array}$$

$\varepsilon_{\alpha \alpha },\varepsilon_{\beta \beta },\varepsilon_{zz},\gamma_{\alpha z},\gamma_{\beta z},\gamma_{\alpha \beta }$ being the strain components within the shell domain which can be found in Ref. [27] (for more details about the displacement field and applications see Ref. [81]).

By applying the displacement components (12), and the strain-displacement relations (13), the strain components can be written as:

(14) $$\begin{array}{l} \left\{\varepsilon_{\alpha \alpha },\varepsilon_{\beta \beta },\varepsilon_{zz},\gamma_{\alpha z},\gamma_{\beta z},\gamma_{\alpha \beta }\right\}=\left\{\varepsilon_{\alpha \alpha }^{0},\varepsilon_{\beta \beta }^{0},\varepsilon_{zz}^{0},\gamma_{\alpha z}^{0},\gamma_{\beta z}^{0},\gamma_{\alpha \beta }^{0}\right\}\\ +z\left\{\varepsilon_{\alpha \alpha }^{1},\varepsilon_{\beta \beta }^{1},\varepsilon_{zz}^{1},\gamma_{\alpha z}^{1},\gamma_{\beta z}^{1},\gamma_{\alpha \beta }^{1}\right\}+z^{2}\left\{\varepsilon_{\alpha \alpha }^{2},\varepsilon_{\beta \beta }^{2},\varepsilon_{zz}^{2},\gamma_{\alpha z}^{2},\gamma_{\beta z}^{2},\gamma_{\alpha \beta }^{2}\right\}\\ +z^{3}\left\{\varepsilon_{\alpha \alpha }^{3},\varepsilon_{\beta \beta }^{3},\varepsilon_{zz}^{3},\gamma_{\alpha z}^{3},\gamma_{\beta z}^{3},\gamma_{\alpha \beta }^{3}\right\} \end{array}$$

with,

(15) $$\begin{array}{l} \left\{\varepsilon_{\alpha \alpha }^{0},\varepsilon_{\beta \beta }^{0},\varepsilon_{zz}^{0},\gamma_{\alpha z}^{0},\gamma_{\beta z}^{0},\gamma_{\alpha \beta }^{0}\right\}=\left\{\frac{\partial u_{0}}{\partial \alpha }+\frac{w_{0}}{R_{1}},\frac{\partial v_{0}}{\partial \alpha }+\frac{w_{0}}{R_{2}},w_{1},\phi_{\alpha }+\frac{\partial w_{0}}{\partial \alpha }-\frac{u_{0}}{R_{1}},\phi_{\beta }+\frac{\partial w_{0}}{\partial \beta }-\frac{v_{0}}{R_{2}},\frac{\partial u_{0}}{\partial \beta }+\frac{\partial v_{0}}{\partial \alpha }\right\};\\ \left\{\varepsilon_{\alpha \alpha }^{1},\varepsilon_{\beta \beta }^{1},\varepsilon_{zz}^{1},\gamma_{\alpha z}^{1},\gamma_{\beta z}^{1},\gamma_{\alpha \beta }^{1}\right\}=\left\{\frac{\partial \phi_{\alpha }}{\partial \alpha }+\frac{w_{1}}{R_{1}},\frac{\partial \phi_{\beta }}{\partial \beta }+\frac{w_{1}}{R_{2}},2w_{2},-\frac{\phi_{\alpha }}{R_{1}},-\frac{\phi_{\beta }}{R_{2}},\frac{\partial \phi_{\alpha }}{\partial \beta }+\frac{\partial \phi_{\beta }}{\partial \alpha }\right\};\\ \left\{\varepsilon_{\alpha \alpha }^{2},\varepsilon_{\beta \beta }^{2},\varepsilon_{zz}^{2},\gamma_{\alpha z}^{2},\gamma_{\beta z}^{2},\gamma_{\alpha \beta }^{2}\right\}=\{-\frac{1}{2}\frac{\partial^{2}w_{1}}{\partial \alpha^{2}}+\frac{w_{2}}{R_{1}},-\frac{1}{2}\frac{\partial^{2}w_{1}}{\partial \beta^{2}}+\frac{w_{2}}{R_{2}},0,3c_{1}\frac{u_{0}}{R_{1}}+3c_{1}c_{3}\frac{\partial w_{2}}{\partial \alpha }+3c_{1}\phi_{\alpha }+\frac{\partial w_{2}}{\partial \alpha }\\ +\frac{1}{2R_{1}}\frac{\partial w_{1}}{\partial \alpha },3c_{1}\frac{v_{0}}{R_{2}}+3c_{1}c_{3}\frac{\partial w_{2}}{\partial \beta }+3c_{1}\phi_{\beta }+\frac{\partial w_{2}}{\partial \beta }+\frac{1}{2R_{1}}\frac{\partial w_{1}}{\partial \beta },-\frac{\partial^{2}w_{1}}{\partial \alpha \partial \beta }\};\\ \left\{\varepsilon_{\alpha \alpha }^{3},\varepsilon_{\beta \beta }^{3},\varepsilon_{zz}^{3},\gamma_{\alpha z}^{3},\gamma_{\beta z}^{3},\gamma_{\alpha \beta }^{3}\right\}=\{c_{1}\frac{\partial^{2}w_{0}}{\partial \alpha^{2}}+c_{1}c_{3}\frac{\partial^{2}w_{2}}{\partial \alpha^{2}}+c_{1}\frac{\partial \phi_{\alpha }}{\partial \alpha },c_{1}\frac{\partial^{2}w_{0}}{\partial \beta^{2}}+c_{1}c_{3}\frac{\partial^{2}w_{2}}{\partial \beta^{2}}+c_{1}\frac{\partial \phi_{\beta }}{\partial \beta },0,c_{1}\frac{\partial^{2}w_{0}}{\partial \beta^{2}}\\ +c_{1}c_{3}\frac{\partial^{2}w_{2}}{\partial \beta^{2}}+c_{1}\frac{\partial \phi_{\beta }}{\partial \beta },\frac{c_{1}}{R_{2}}\frac{\partial w_{0}}{\partial \beta }+\frac{c_{1}c_{3}}{R_{2}}\frac{\partial w_{2}}{\partial \beta }+\frac{c_{1}}{R_{2}}\phi_{\beta },2c_{1}\frac{\partial^{2}w_{0}}{\partial \alpha \partial \beta }+2c_{1}c_{2}\frac{\partial^{2}w_{2}}{\partial \alpha \partial \beta }+c_{1}\frac{\partial \phi_{\alpha }}{\partial \beta }+c_{1}\frac{\partial \phi_{\beta }}{\partial \alpha }\} \end{array}$$

The Hamilton’s principle is applied here to determine the governing equations, namely:

(16) $$\int_{0}^{t}\delta \left(U-T+W\right)dt=0$$

where U,T, and W stands for the strain energy, kinetic energy and external work, respectively. More specifically, the strain energy can be expressed as:

(17) $$\delta U=\int_{v}\left(\sigma_{ij}\delta \varepsilon_{ij}\right)dV=\int_{v}\left(\sigma_{\alpha \alpha }\delta \varepsilon_{\alpha \alpha }+\sigma_{\beta \beta }\delta \varepsilon_{\beta \beta }+\sigma_{zz}\delta \varepsilon_{zz}+\tau_{\beta z}\delta \gamma_{\beta z}+\tau_{\alpha z}\delta \gamma_{\alpha z}+\tau_{\alpha \beta }\delta \gamma_{\alpha \beta }\right)dV$$

More details about this Equation (17) can be found in Appendix A. Moreover, the variation of the kinetic energy obtains

(18) δK=∬R∫−h2h2ρuiδuidV=∫0a∫0b∫−h2h2ρ∂ui∂tδ∂ui∂tdzdαdβ

For more details, see Appendix B. The external work related to the applied forces can be defined as:

(19) $$\delta W=\int_{0}^{L}\left[f_{\mathrm{hygrothermal}}\right]d\alpha$$

where the hygrothermal forces $f_{\mathrm{hygrothermal}}$ are expressed as [82,83,84]:

(20) $$f_{\mathrm{hygrothermal}}=(f_{\mathrm{thermal}}+f_{\mathrm{hygro}})\delta \nabla^{2}w$$

Including both the temperature T and moisture concentration H, i.e.,

(21) $$\begin{array}{l} f_{\mathrm{thermal}}=\int_{-\frac{h}{2}}^{\frac{h}{2}}\frac{E(z)}{1-\nu }\alpha (z)(T_{c}-T_{m})dz\\ f_{\mathrm{hygro}}=\int_{-\frac{h}{2}}^{\frac{h}{2}}\int_{-\frac{h}{2}}^{\frac{h}{2}}\frac{E(z)}{1-\nu }\beta (z)(H_{c}-H_{m})dz \end{array}$$

Considering the influence of the environmental conditions on the mechanical properties of the nanostructures is a key point for structural analyses. More specifically, the environmental conditions may include wet-dry, freeze-thaw, sea-water and also moisture impression. Among them, the temperature rise and absorption of moisture represent the most popular conditions typically explored for FGMs. 

By combining Equations (17), (18), (21) with Equation (16), the Euler–Lagrange equations can be obtained, while assuming the coefficients of $\delta u,\delta v,\delta w_{0},\delta \phi_{\alpha },\delta \phi_{\beta },\delta w_{1},\;\mathrm{and}\;\delta w_{2}$ equal to zero. Additional information about the mathematical manipulation are included in Appendix C. Thus, we are able to show the partial high-order non-local strain gradient shear deformable equation via displacements by inserting Equations (A5)–(A11) into Equation (8).

It is worth mentioning that shell models for nanomaterials can be used for studying important nanostructures such as nanotubes and spherical nanoparticles. Considering the wide applications of these nanostructures, an accurate modeling is required to predict their structural behavior, as found in the literature for medical applications, energy and environment applications, thermal interfaces, solar cells, coating, spaceflight applications, and fluorescent sensors [85,86,87,88,89]. A practical example of the methodology proposed in this work can be found in [90,91] for calculating small scale parameters which are related to different factors at an atomic level of nano-scaled materials. A consistency check of the proposed formulation was proposed in [92] to predict the material properties of nanocomposites, while comparing the theoretical results in terms of wave propagation with the experimental predictions based on some non-destructive tests.

## 3. Analytical Wave Propagation Solution

In what follows, we investigate the bulk waves. This means that the waves propagate far from any boundary zone. Using a harmonic method for nanoshells made of porous materials, we approximate the solution as:

(22) $$\begin{array}{l} u_{0}=A_{1}\exp\left(i\alpha k_{\alpha }+i\beta k_{\beta }-i\omega t\right);\;v_{0}=A_{2}\exp\left(i\alpha k_{\alpha }+i\beta k_{\beta }-i\omega t\right);\\ w_{0}=A_{3}\exp\left(i\alpha k_{\alpha }+i\beta k_{\beta }-i\omega t\right);\;\\ \phi_{\alpha }=A_{4}\exp\left(i\alpha k_{\alpha }+i\beta k_{\beta }-i\omega t\right);\phi_{\beta }=A_{5}\exp\left(i\alpha k_{\alpha }+i\beta k_{\beta }-i\omega t\right);\\ w_{1}=A_{6}\exp\left(i\alpha k_{\alpha }+i\beta k_{\beta }-i\omega t\right);w_{2}=A_{7}\exp\left(i\alpha k_{\alpha }+i\beta k_{\beta }-i\omega t\right) \end{array}$$

where $k_{\alpha }$ and $k_{\beta }$ denote the wave numbers along α− and β− directions, respectively; ω is the circular frequency; $i=\sqrt{-1}$ and $A_{1}-A_{7}$ are the wave amplitudes.

By inserting Equation (31) into partial differential non-local strain gradient equations, and collecting the coefficients, the following characteristic equations are obtained:

(23) $$\left(K-\omega^{2}M\right)X=0$$

in which K and M stand for the stiffness and mass matrixes, respectively; and the eigenvector can be written as $X={\left\{A_{1},A_{2},A_{3},A_{4},A_{5},A_{6},A_{7}\right\}}^{T}$. Once the circular frequency ω is computed, the phase velocity can be easily given by c=ω/k, with $k=k_{\alpha }=k_{\beta }$.

## 4. Numerical Results and Discussion

In this study, we combine the bi-Helmholtz non-local theory and strain gradient theory to study the wave propagation in doubly-curved nanoshells made of P-FGMs, immersed in a hygrothermal environment. The material properties of the FG nanoshells are summarized in Table 1. In what follows, the thickness of the nanoshell is set to *h* = 2 nm.

**Figure 3:**
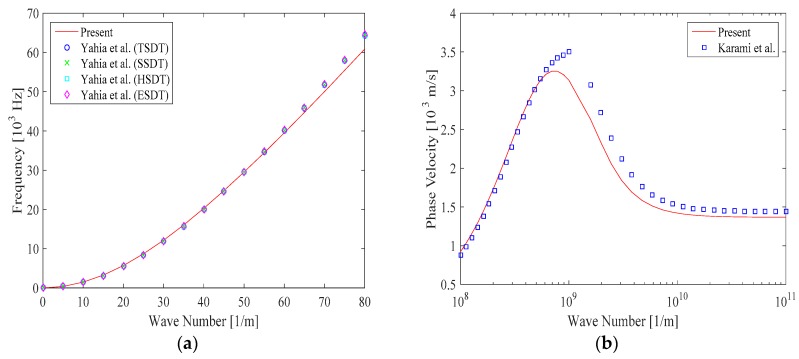
Comparison of wave frequency in rectangular plate (**a**) and phase velocity in P-FGM nanoplates (**b**) vs. the wave number. $\left(l=0.2,\mu_{0}=\mu_{1}=\mu =1,\;n=1,\;\xi =0.2\right)$.

First, we check for the efficiency and accuracy of the proposed approach for the selected problem. Due to the authors’ best knowledge, there is a general lack of works in the open literature in which the size-dependent wave propagation of P-FGM doubly-curved nanoshells was investigated. Only in the limit case of infinite radius of curvature, the doubly-curved nanoshell would revert to the nanoplate. Thus, some different mathematical models from the literature are here selected for comparative purposes. More specifically, our results are evaluated in terms of wave frequency in a comparative way with predictions by Yahia et al. [42] based on different HSDTs. The size-dependent phase velocity is also evaluated and compared to the results predicted by Karami et al. [71] via the non-local strain gradient second-order shear deformation plate theory. As seen in Figure 3, it is worth noticing a very good agreement between the results which confirms the accuracy of the method and solution here proposed in this work.

**Figure 4:**
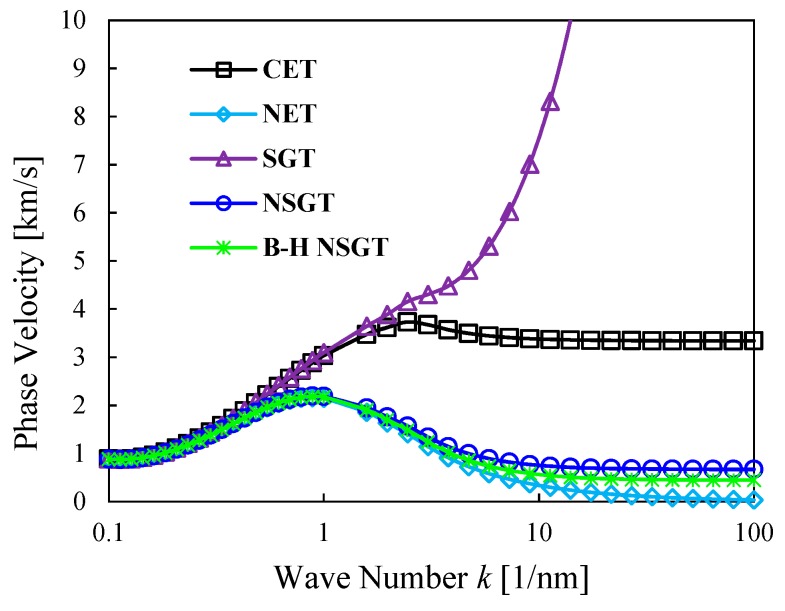
Phase velocity of the P-FGM doubly-curved nanoshell vs. the wave number for different elasticity theories, $\mu_{0}=\mu_{1}=\mu =0,\;\mathrm{and}\;l=0$ for Classical Elasticity Theory (CET); $\mu_{0}=\mu_{1}=\mu =1.0\;\mathrm{nm},\;\mathrm{and}\;l=0$ for non-local elasticity theory (NET); $\mu_{0}=\mu_{1}=\mu =0\;,\;\mathrm{and}\;l=0.2\;\mathrm{nm}$ for strain gradient theory (SGT); $\mu_{0}=\mu_{1}=\mu =1.0\;\mathrm{nm},\;\mathrm{and}\;l=0.2\;\mathrm{nm}$ for non-local strain gradient theory (NSGT); $\mu_{0}=1\;\mathrm{nm},\;\mu_{1}=1.5\;\mathrm{nm},\mathrm{and}\;l=0.2\;\mathrm{nm}$ for the B-H NSGT. (*R*_2_ = *R*_1_ = 50 × *h*, *n* = 2, *ξ* = 0.1).

A further comparative evaluation of the results is plotted in Figure 4, in terms of flexural dispersion relation among the phase velocity and wave number. Here we compare the results based on the B-H NSGT with different continuum theories, namely, the Classical Elasticity Theory (CET); the Non-local Elasticity Theory (NET); the Strain Gradient Theory (SGT); and the Non-local Strain Gradient Theory (NSGT). The phase velocity derived from all continuum theories increases with the increase of the wave number, at least for low wave numbers. It is noteworthy that, the flexural dispersion relations calculated using all continuum theories are almost identical when the wave number is very small (almost k≤0.51/nm). This means that all theories give reliable results whenever the wave number is very small. By contrast, an increased wave number, leads to different trends depending on the selected continuum theory. The phenomenon is also detected in terms of transverse waves in a CNT [93], longitudinal waves in axial bars [94] and transverse and longitudinal waves in FG plates [95]. Based on Figure 4, it seems that the phase velocities of CET and SGT are larger than NSGT, while the phase velocities of NET and B-H NSGT are smaller than those based on NSGT. In addition, the phase velocities always increase with increased wave numbers for the SGT case.

**Figure 5:**
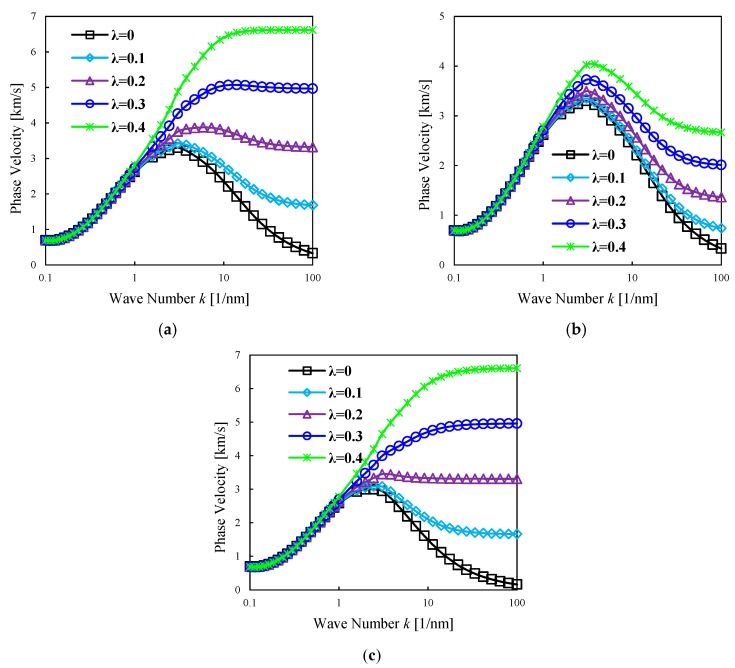
Phase velocity of FG doubly-curved nanoshell vs. the wave number for different non-local and strain gradient parameters. $R_{2}=R_{1}=50\times h,n=2,\;\xi =0.1$; (**a**) $\mu_{0}=0.1,\;\mu_{1}=0.2$, (**b**) $\mu_{0}=0.1,\;\mu_{1}=0.5$, (**c**) $\mu_{0}=0.2,\;\mu_{1}=0.2$.

To illustrate the effect of the strain gradient length scale parameter and lower/higher order non-local parameters, the flexural dispersion curves for phase velocity of doubly-curved nanoshells made of P-FGMs is plotted in Figure 5 with respect to wave number. The power-law index is set to *n =* 1 and the porosity volume fraction is kept constant to ξ=0.1. Typically, the phase velocity increases for increasing wave numbers up to a maximum value. According to the model here proposed, after a certain value of the wave number, the phase velocity depends on the small-scale parameters. It seems also that the strain gradient size-dependence does not have an adequate effect on the wave characteristics when the values of wave number is small, whereby the characteristics of waves is highly affected at large wave numbers. 

Moreover, the strain gradient size-dependence is sensibly smaller than the non-local one, and the phase velocity decreases for increasing wave numbers after the peak value. However, when the strain gradient parameter is larger than the non-local parameter, an increased wave number yields larger phase velocities. Another important result is that a higher order non-local parameter (*μ_1_*) reduces its effect on phase velocities compared to the lower order non-local parameter (*μ_0_*). This means that it is very important to consider two non-local parameters and one strain gradient length scale parameter for propagation studies of nanostructures.

**Figure 6:**
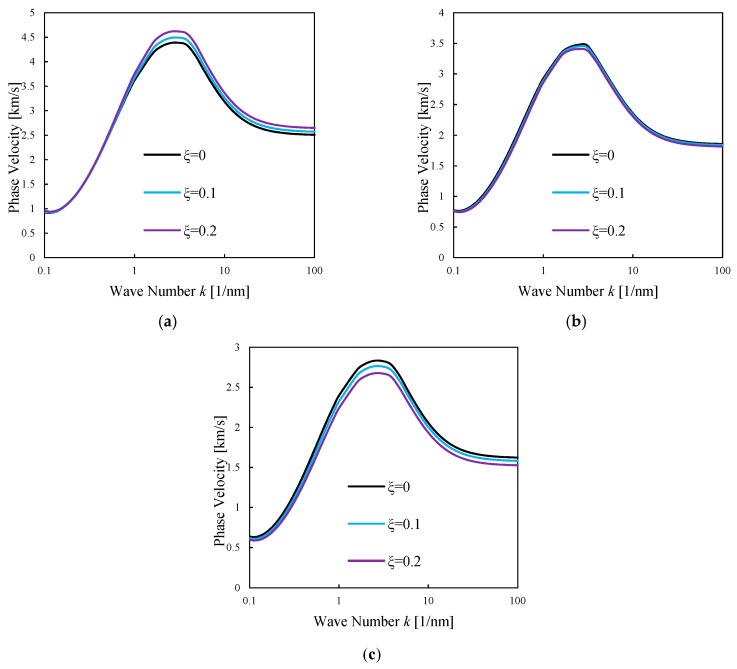
Phase velocity of P-FGM doubly-curved nanoshell vs. the wave number for different material compositions and porosity coefficients. $R_{2}=R_{1}=50\times h,\;\lambda =0.1,\;\mu_{0}=\mu_{1}=0.2$; (**a**) *n* = 0.2, (**b**) *n* = 1, (**c**) *n* = 5.

To consider the porosity and material compositions effects on the wave characteristics of doubly-curved nanoshells made of P-FGMs, Figure 6 plots the flexural dispersion curves for phase velocities under the aforementioned effects vs. the wave number. As visible in Figure 6, the porosity effect depends significantly on the value of the selected power-law index. In detail, an incremental effect of porosity is reversed by increasing the power-law index. In other words, porosity in composite materials with higher percentages of ceramic phase yields an increased value of the phase velocities. Instead, the porosity in composite materials with a higher percentages of metal phase yields a decreased value of the phase velocities in nanoshell structures. The power-law index shows a decreased effect on the phase velocity of FGM-based nanoshells for each wave number.

**Figure 7:**
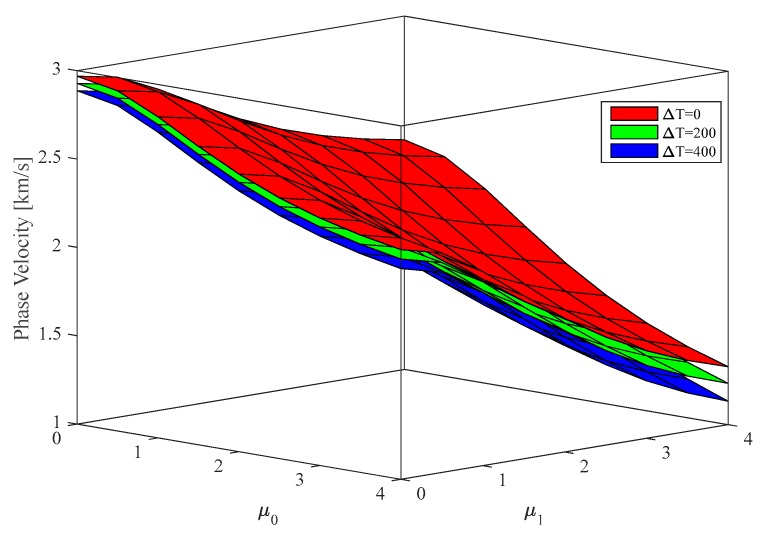
Influence of temperature on the phase velocity of P-FGM doubly-curved nanoshell vs. lower/higher order non-local parameters. $\left(k=5\times 10^{8}\;,\;R_{2}=R_{1}=50\times h,\;n=2,\;\xi =0.1,\;\lambda =2\right)$.

**Figure 8:**
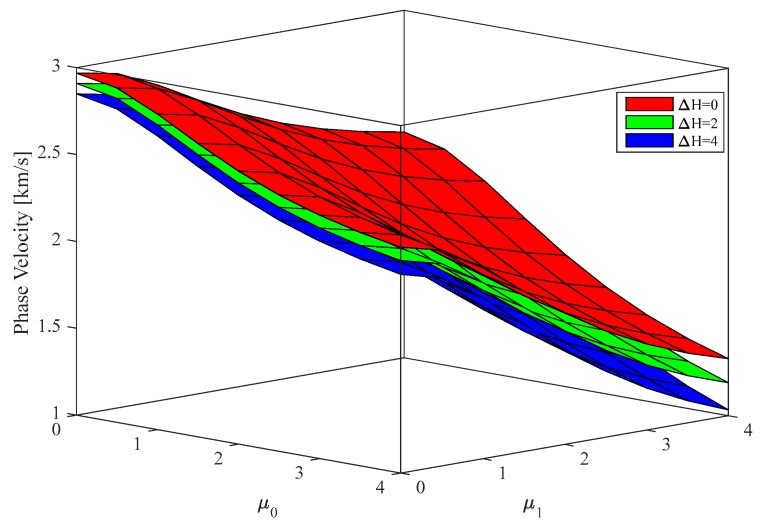
Influence of humidity on the phase velocity of P-FGM doubly-curved nanoshell vs. lower/higher order non-local parameters. $\left(k=5\times 10^{8}\;,\;R_{2}=R_{1}=50\times h,\;n=2,\;\xi =0.1,\;\lambda =2\right)$.

To survey the hygrothermal environment, Figure 7 and Figure 8 show the effect of a varying temperature and humidity on the phase velocity of P-FGM nanoshells, while keeping fixed the following parameters $k=5\times 10^{8}\;,\;R_{2}=R_{1}=50\times h,\;n=2,\;\xi =0.1,\;\lambda =2$. As expected, an increasing temperature and humidity reduces the phase velocity of a P-FGM nanoshell. Moreover, at a prescribed environmental condition, the phase velocities decrease for increasing values of non-local parameters.

**Figure 9:**
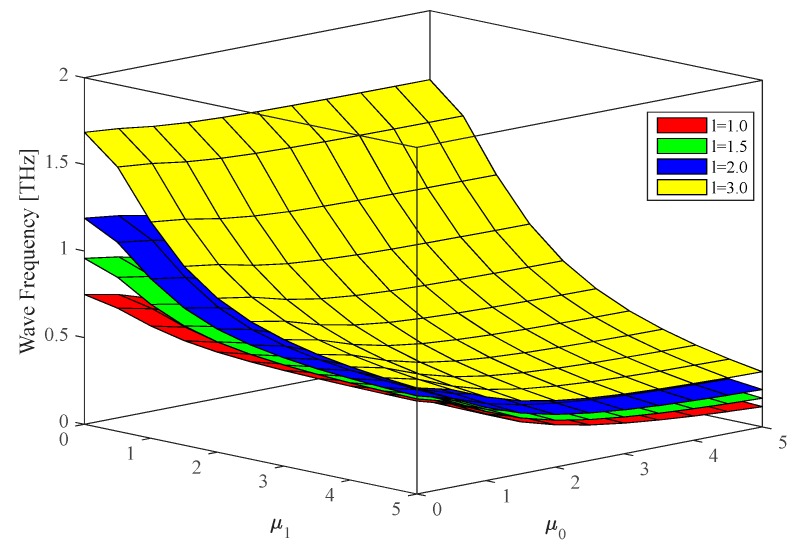
Variation of the wave frequency in P-FGM doubly-curved nanoshells vs. lower/higher order non-local parameters. $\left(R_{2}=R_{1}=50\times h,\;n=1,\;\xi =0.2,\;\Delta T=100,\;\Delta H=2\right)$.

The last parametric investigation analyses the effect of lower- higher order non-local parameters variations on the wave frequency of FGMs nanoshells in a hygrothermal environment, while assuming $R_{2}=R_{1}=50\times h,\;n=1,\;\xi =0.2,$ ΔT=100, ΔH=2. The phase velocities decrease with the double increase of both non-local parameters (see Figure 9). Higher-order non-local parameters feature a more pronounced effect on the wave propagation of P-FGM nanoshells compared to the non-local parameters of a lower order.

## 5. Conclusions

This work analyzes the size-dependent wave propagation of doubly-curved nanoshells made of P-FGMs, exposed to a hygrothermal environment including both the hardening and softening stiffnesses. To this end, a high order non-local strain gradient theory of elasticity was incorporated to a HSDT theory including the stretching effects. Hamilton’s principle is here applied to determine the governing equations of the problem, solved analytically here to determine the wave frequencies of the nanostructures. Based on a large parametric investigation, the main conclusions can be summarized as follows:

(1) The non-local strain gradient model (NSGT), applied here for porous nanoshells, provides the same results as those ones obtained for porous nanoplates, nanobeams and nanotubes.
(2) The hygrothermal environment has a decreasing effect on the variation of the phase velocity at all small-scale parameters.
(3) The porosity has varying effect on the results of nanoshells depending on the selected value of the power-lax index parameter. This conclusion is perfectly in line with similar findings from the literature obtained for nanobeams and nanotubes.
(4) The effect of small-scale parameters on the structural response of nanostructures depends on the wave number value and the nature of the selected material.
(5) The geometry does not affect significantly the final trend of results. Thus, it is suggested to use the most simplified structures such as the beam and tube, instead of plates and shells, when the main focus of the analysis is just related to the size-dependent behavior of a nanostructure system. In this way, the study of possible size-dependent effects could disregard any kind of complex equations.

## Figures and Tables

**Table 1:** Material properties of the used ${(\mathrm{Al}/\mathrm{Al}}_{2}O_{3})$ functionally graded (FG) doubly-curved nanoshell.

Material	*E *(GPa)	$\rho \;(\mathrm{kg}/m^{3})$	ν	α (/K)	$\beta {\left(\mathrm{wt}{.\;\%H}_{2}O\right)}^{-1}$
Aluminum (Al)	70	2702	0.3	$23\times 10^{-6}$	0.44
$\mathrm{Alumina}\;{(\mathrm{Al}}_{2}O_{3})$	380	3800	0.3	$7\times 10^{-6}$	0.001

## Appendix A

(A1) $$\begin{array}{l} \delta U=\int_{0}^{a}\int_{0}^{b}[N_{\alpha \alpha }(\frac{\partial \delta u_{0}}{\partial \alpha }+\frac{1}{R_{1}}\delta w_{0})+M_{\alpha \alpha }(\frac{\partial \delta \phi_{\alpha }}{\partial \alpha }+\frac{1}{R_{1}}\delta w_{1})+O_{\alpha \alpha }(-\frac{1}{2}\frac{\partial^{2}\delta w_{1}}{\partial \alpha^{2}}+\frac{1}{R_{1}}\delta w_{2})\\ +P_{\alpha \alpha }(c_{1}\frac{\partial^{2}\delta w_{0}}{\partial \alpha^{2}}+c_{1}\frac{\partial \delta \phi_{\alpha }}{\partial \alpha }+c_{1}c_{3}\frac{\partial^{2}\delta w_{2}}{\partial \alpha^{2}})+N_{\beta \beta }(\frac{\partial \delta v_{0}}{\partial \beta }+\frac{1}{R_{2}}\delta w_{0})+M_{\beta \beta }(\frac{\partial \delta \phi_{\beta }}{\partial \beta }+\frac{1}{R_{2}}\delta w_{1})\\ +O_{\beta \beta }(-\frac{1}{2}\frac{\partial^{2}\delta w_{1}}{\partial \beta^{2}}+\frac{1}{R_{2}}\delta w_{2})+P_{\beta \beta }(c_{1}\frac{\partial^{2}\delta w_{0}}{\partial \beta^{2}}+c_{1}\frac{\partial \delta \phi_{\beta }}{\partial \beta }+c_{1}c_{3}\frac{\partial^{2}\delta w_{2}}{\partial \beta^{2}})+N_{zz}\delta w_{1}+2M_{zz}\delta w_{2}\\ +N_{\beta z}(\frac{1}{R_{2}}\delta v_{0}+\frac{\partial \delta w_{0}}{\partial \beta }+\delta \phi_{\beta })-\frac{1}{R_{2}}M_{\beta z}\delta \phi_{\beta }+O_{\beta z}(3c_{1}\frac{\partial \delta w_{0}}{\partial \beta }+3c_{1}\delta \phi_{\beta }+\frac{1}{2R_{2}}\frac{\partial \delta w_{1}}{\partial \beta })\\ -P_{\beta z}(\frac{c_{1}}{R_{2}}\frac{\partial \delta w_{0}}{\partial \beta }+\frac{c_{1}}{R_{2}}\delta \phi_{\beta }+\frac{c_{1}c_{3}}{R_{2}}\frac{\partial \delta w_{2}}{\partial \beta })+N_{\alpha z}(\frac{1}{R_{1}}\delta u_{0}+\frac{\partial \delta w_{0}}{\partial \alpha }+\delta \phi_{\alpha })-\frac{1}{R_{1}}M_{\alpha z}\delta \phi_{\alpha }\\ +O_{\alpha z}(3c_{1}\frac{\partial \delta w_{0}}{\partial \alpha }+3c_{1}\delta \phi_{\alpha }+\frac{1}{2R_{1}}\frac{\partial \delta w_{1}}{\partial \alpha })-P_{\alpha z}(\frac{c_{1}}{R_{1}}\frac{\partial \delta w_{0}}{\partial \alpha }+\frac{c_{1}}{R_{1}}\delta \phi_{\alpha }+\frac{c_{1}c_{3}}{R_{1}}\frac{\partial \delta w_{2}}{\partial \alpha })+N_{\alpha \beta }(\frac{\partial \delta u_{0}}{\partial \beta }+\frac{\partial \delta v_{0}}{\partial \alpha })\\ +M_{\alpha \beta }(\frac{\partial \delta \phi_{\alpha }}{\partial \beta }+\frac{\partial \delta \phi_{\beta }}{\partial \alpha })-O_{\alpha \beta }\frac{\partial^{2}\delta w_{1}}{\partial \alpha \partial \beta }+P_{\alpha \beta }(2c_{1}\frac{\partial^{2}\delta w_{0}}{\partial \alpha \partial \beta }+c_{1}\frac{\partial \delta \phi_{\alpha }}{\partial \beta }+c_{1}\frac{\partial \delta \phi_{\beta }}{\partial \alpha }+2c_{1}c_{3}\frac{\partial^{2}\delta w_{2}}{\partial \alpha \partial \beta })]d\beta d\alpha \end{array}$$

where,

(A2) $$\left[\begin{array}{cccc} N_{\alpha \alpha } & M_{\alpha \alpha } & O_{\alpha \alpha } & P_{\alpha \alpha }\\ N_{\beta \beta } & M_{\beta \beta } & O_{\beta \beta } & P_{\beta \beta }\\ N_{zz} & M_{zz} & O_{zz} & P_{zz}\\ N_{\beta z} & M_{\beta z} & O_{\beta z} & P_{\beta z}\\ N_{\alpha z} & M_{\alpha z} & O_{\alpha z} & P_{\alpha z}\\ N_{\alpha \beta } & M_{\alpha \beta } & O_{\alpha \beta } & P_{\alpha \beta } \end{array}\right]=\int_{-\frac{h}{2}}^{\frac{h}{2}}\left\{\begin{array}{l} \sigma_{\alpha \alpha }\\ \sigma_{\beta \beta }\\ \sigma_{zz}\\ \tau_{\alpha z}\\ \tau_{\beta z}\\ \tau_{\alpha \beta } \end{array}\right\}\left(1,z,z^{2},z^{3}\right)dz$$

## Appendix B

(A3) $$\begin{array}{l} \delta K=\int_{0}^{a}\int_{0}^{b}[I_{0}(\frac{\partial u_{0}}{\partial t}\frac{\partial \delta u_{0}}{\partial t}+\frac{\partial v_{0}}{\partial t}\frac{\partial \delta v_{0}}{\partial t}+\frac{\partial w_{0}}{\partial t}\frac{\partial \delta w_{0}}{\partial t})+I_{1}(\frac{\partial u_{0}}{\partial t}\frac{\partial \delta \phi_{\alpha }}{\partial t}+\frac{\partial v_{0}}{\partial t}\frac{\partial \delta \phi_{\beta }}{\partial t}+\frac{\partial w_{0}}{\partial t}\frac{\partial \delta w_{1}}{\partial t}\\ +\;\frac{\partial \phi_{\alpha }}{\partial t}\frac{\partial \delta u_{0}}{\partial t}\;+\;\frac{\partial \phi_{\beta }}{\partial t}\frac{\partial \delta v_{0}}{\partial t}\;+\;\frac{\partial w_{1}}{\partial t}\frac{\partial \delta w_{0}}{\partial t})+\;I_{2}(-\frac{1}{2}\frac{\partial u_{0}}{\partial t}\frac{\partial^{2}\delta w_{1}}{\partial \alpha \partial t}-\;\frac{1}{2}\frac{\partial v_{0}}{\partial t}\frac{\partial^{2}\delta w_{1}}{\partial \beta \partial t}\;+\frac{\partial w_{0}}{\partial t}\frac{\partial \delta w_{2}}{\partial t}\\ +\frac{\partial \phi_{\alpha }}{\partial t}\frac{\partial \delta \phi_{\alpha }}{\partial t}+\frac{\partial \phi_{\beta }}{\partial t}\frac{\partial \delta \phi_{\beta }}{\partial t}-\frac{1}{2}\frac{\partial^{2}w_{1}}{\partial \alpha \partial t}\frac{\partial \delta u_{0}}{\partial t}-\frac{1}{2}\frac{\partial^{2}w_{1}}{\partial \alpha \partial t}\frac{\partial \delta v_{0}}{\partial t}+\frac{\partial w_{1}}{\partial t}\frac{\partial \delta w_{1}}{\partial t}+\;\frac{\partial w_{2}}{\partial t}\frac{\partial \delta w_{0}}{\partial t})\\ +I_{3}(\frac{\partial u_{0}}{\partial t}(c_{1}\frac{\partial^{2}\delta w_{0}}{\partial \alpha \partial t}+c_{1}c_{3}\frac{\partial^{2}\delta w_{2}}{\partial \alpha \partial t}+c_{1}\frac{\partial \delta \phi_{\alpha }}{\partial t})+\frac{\partial v}{\partial t}(c_{1}\frac{\partial^{2}\delta w_{0}}{\partial \beta \partial t}+c_{1}c_{3}\frac{\partial^{2}\delta w_{2}}{\partial \beta \partial t}+c_{1}\frac{\partial \delta \phi_{\beta }}{\partial t})\\ +c_{1}\frac{\partial^{2}w_{0}}{\partial \alpha \partial t}\frac{\partial \delta u_{0}}{\partial t}+c_{1}\frac{\partial^{2}w_{0}}{\partial \beta \partial t}\frac{\partial \delta v_{0}}{\partial t}\;-\;\frac{1}{2}\frac{\partial \phi_{\alpha }}{\partial t}\frac{\partial^{2}\delta w_{1}}{\partial \alpha \partial t}\;+c_{1}\frac{\partial \phi_{\alpha }}{\partial t}\frac{\partial \delta u_{0}}{\partial t}\;-\frac{1}{2}\frac{\partial \phi_{\beta }}{\partial t}\frac{\partial^{2}\delta w_{1}}{\partial \beta \partial t}\;+c_{1}\frac{\partial \phi_{\beta }}{\partial t}\frac{\partial \delta v_{0}}{\partial t}\\ -\frac{1}{2}\frac{\partial^{2}w_{1}}{\partial \alpha \partial t}\frac{\partial \delta \phi_{\alpha }}{\partial t}\frac{\partial w_{1}}{\partial t}\frac{\partial \delta w_{2}}{\partial t}-\frac{1}{2}\frac{\partial^{2}w_{2}}{\partial \beta \partial t}\frac{\partial \delta \phi_{\beta }}{\partial t}+c_{1}c_{3}\frac{\partial^{2}w_{2}}{\partial \alpha \partial t}\frac{\partial \delta u_{0}}{\partial t}+c_{1}c_{3}\frac{\partial^{2}w_{2}}{\partial \beta \partial t}\frac{\partial \delta v_{0}}{\partial t}+\frac{\partial w_{2}}{\partial t}\frac{\partial \delta w_{1}}{\partial t})\\ +I_{4}(c_{1}\frac{\partial^{2}w_{0}}{\partial \alpha \partial t}\frac{\partial \delta \phi_{\alpha }}{\partial t}+c_{1}\frac{\partial^{2}w_{0}}{\partial \beta \partial t}\frac{\partial \delta \phi_{\beta }}{\partial t}+\frac{\partial \phi_{\alpha }}{\partial t}(c_{1}\frac{\partial^{2}\delta w_{0}}{\partial \alpha \partial t}+c_{1}c_{3}\frac{\partial^{2}\delta w_{2}}{\partial \alpha \partial t}+c_{1}\frac{\partial \delta \phi_{\alpha }}{\partial t})+c_{1}\frac{\partial \phi_{\alpha }}{\partial t}\frac{\partial \delta \phi_{\alpha }}{\partial t}\\ +\frac{\partial \phi_{\beta }}{\partial t}(c_{1}\frac{\partial^{2}\delta w_{0}}{\partial \beta \partial t}+c_{1}c_{3}\frac{\partial^{2}\delta w_{2}}{\partial \beta \partial t}+c_{1}\frac{\partial \delta \phi_{\beta }}{\partial t})+c_{1}\frac{\partial \phi_{\beta }}{\partial t}\frac{\partial \delta \phi_{\beta }}{\partial t}\;+\frac{1}{4}\frac{\partial^{2}w_{1}}{\partial \alpha \partial t}\frac{\partial^{2}\delta w_{1}}{\partial \alpha \partial t}\;+\frac{1}{4}\frac{\partial^{2}w_{1}}{\partial \beta \partial t}\frac{\partial^{2}\delta w_{1}}{\partial \beta \partial t}\\ +c_{1}c_{3}\frac{\partial^{2}w_{2}}{\partial \alpha \partial t}\frac{\partial \phi_{\alpha }}{\partial t}+c_{1}c_{3}\frac{\partial^{2}w_{2}}{\partial \beta \partial t}\frac{\partial \phi_{\beta }}{\partial t})+I_{5}(-\frac{c_{1}}{2}\frac{\partial^{2}w_{0}}{\partial \alpha \partial t}\frac{\partial^{2}\delta w_{1}}{\partial \alpha \partial t}-\frac{c_{1}}{2}\frac{\partial^{2}w_{0}}{\partial \beta \partial t}\frac{\partial^{2}\delta w_{1}}{\partial \beta \partial t}-\frac{c_{1}}{2}\frac{\partial \phi_{\alpha }}{\partial t}\frac{\partial^{2}\delta w_{1}}{\partial \alpha \partial t}\\ -\frac{c_{1}}{2}\frac{\partial \phi_{\beta }}{\partial t}\frac{\partial^{2}\delta w_{1}}{\partial \beta \partial t}-\frac{1}{2}\frac{\partial^{2}w_{1}}{\partial \alpha \partial t}(c_{1}\frac{\partial^{2}\delta w_{0}}{\partial \alpha \partial t}+c_{1}c_{3}\frac{\partial^{2}\delta w_{2}}{\partial \alpha \partial t}+c_{1}\frac{\partial \delta \phi_{\alpha }}{\partial t})-\frac{1}{2}\frac{\partial^{2}w_{1}}{\partial \beta \partial t}(c_{1}\frac{\partial^{2}\delta w_{0}}{\partial \beta \partial t}+c_{1}\frac{\partial \delta \phi_{\beta }}{\partial t}\\ +c_{1}c_{3}\frac{\partial^{2}\delta w_{2}}{\partial \beta \partial t})-\frac{c_{1}c_{3}}{2}\frac{\partial^{2}w_{2}}{\partial \alpha \partial t}\frac{\partial^{2}\delta w_{1}}{\partial \alpha \partial t}-\frac{c_{1}c_{3}}{2}\frac{\partial^{2}w_{2}}{\partial \beta \partial t}\frac{\partial^{2}\delta w_{1}}{\partial \beta \partial t})+I_{6}(c_{1}\frac{\partial^{2}w_{0}}{\partial \alpha \partial t}(c_{1}\frac{\partial^{2}\delta w_{0}}{\partial \alpha \partial t}+c_{1}c_{3}\frac{\partial^{2}\delta w_{2}}{\partial \alpha \partial t}\\ +c_{1}\frac{\partial \delta \phi_{\alpha }}{\partial t})+c_{1}\frac{\partial^{2}w_{0}}{\partial \beta \partial t}\;(c_{1}\frac{\partial^{2}\delta w_{0}}{\partial \beta \partial t}\;+c_{1}c_{3}\frac{\partial^{2}\delta w_{2}}{\partial \beta \partial t}+c_{1}\frac{\partial \delta \phi_{\beta }}{\partial t})\;+\;c_{1}\frac{\partial \phi_{\alpha }}{\partial t}\;(c_{1}\frac{\partial^{2}\delta w_{0}}{\partial \alpha \partial t}+c_{1}c_{3}\frac{\partial^{2}\delta w_{2}}{\partial \alpha \partial t}\\ +c_{1}\frac{\partial \delta \phi_{\alpha }}{\partial t})+c_{1}\frac{\partial \phi_{\beta }}{\partial t}(c_{1}\frac{\partial^{2}\delta w_{0}}{\partial \beta \partial t}+c_{1}c_{3}\frac{\partial^{2}\delta w_{2}}{\partial \beta \partial t}+c_{1}\frac{\partial \delta \phi_{\beta }}{\partial t})+c_{1}c_{3}\frac{\partial^{2}w_{2}}{\partial \alpha \partial t}(c_{1}\frac{\partial^{2}\delta w_{0}}{\partial \alpha \partial t}+c_{1}c_{3}\frac{\partial^{2}\delta w_{2}}{\partial \alpha \partial t}\\ +c_{1}\frac{\partial \delta \phi_{\alpha }}{\partial t})+c_{1}c_{3}\frac{\partial^{2}w_{2}}{\partial \beta \partial t}(c_{1}\frac{\partial^{2}\delta w_{0}}{\partial \beta \partial t}+c_{1}c_{3}\frac{\partial^{2}\delta w_{2}}{\partial \beta \partial t}+c_{1}\frac{\partial \delta \phi_{\beta }}{\partial t}))]d\beta d\alpha \end{array}$$

where,

(A4) $$\left\{I_{0},I_{1},I_{2},I_{3},I_{4},I_{5},I_{6}\right\}=\int_{-\frac{h}{2}}^{\frac{h}{2}}\rho \left(1,z,z^{2},z^{3},z^{4},z^{5},z^{6}\right)dz$$

## Appendix C

(A5) $$\begin{array}{l} -\frac{\partial N_{\alpha \alpha }}{\partial \alpha }-\frac{N_{\alpha z}}{R_{1}}-\frac{\partial N_{\alpha \beta }}{\partial \beta }=-I_{0}\frac{\partial^{2}u_{0}}{\partial t^{2}}-I_{1}\frac{\partial^{2}\phi_{\alpha }}{\partial t^{2}}+\frac{1}{2}I_{2}\frac{\partial^{3}w_{1}}{\partial \alpha \partial t^{2}}-c_{1}I_{3}\frac{\partial^{3}w_{0}}{\partial \alpha \partial t^{2}}\\ -c_{1}c_{3}I_{3}\frac{\partial^{2}\phi_{\alpha }}{\partial t^{2}}-c_{1}c_{3}I_{3}\frac{\partial^{3}w_{2}}{\partial \alpha \partial t^{2}} \end{array}$$

(A6) $$\begin{array}{l} -\frac{\partial N_{\beta \beta }}{\partial \beta }-\frac{N_{\beta z}}{R_{2}}-\frac{\partial N_{\alpha \beta }}{\partial \alpha }=-I_{0}\frac{\partial^{2}v_{0}}{\partial t^{2}}-I_{1}\frac{\partial^{2}\phi_{\beta }}{\partial t^{2}}+\frac{1}{2}I_{2}\frac{\partial^{3}w_{1}}{\partial \beta \partial t^{2}}-c_{1}I_{3}\frac{\partial^{3}w_{0}}{\partial \beta \partial t^{2}}\\ -c_{1}I_{3}\frac{\partial^{2}\phi_{\beta }}{\partial t^{2}}-c_{1}c_{3}I_{3}\frac{\partial^{3}w_{2}}{\partial \beta \partial t^{2}} \end{array}$$

(A7) $$\begin{array}{l} \frac{N_{\alpha \alpha }}{R_{1}}\;+\;c_{1}\frac{\partial^{2}P_{\alpha \alpha }}{\partial \alpha^{2}}\;+\;\frac{N_{\beta \beta }}{R_{2}}\;+\;c_{1}\frac{\partial^{2}P_{\beta \beta }}{\partial \beta^{2}}\;-\;\frac{\partial N_{\beta z}}{\partial \beta }\;-3c_{1}\frac{\partial O_{\beta z}}{\partial \beta }\;+\frac{c_{1}}{R_{2}}\frac{\partial P_{\beta z}}{\partial \beta }\;\\ -\frac{\partial N_{\alpha z}}{\partial \alpha }\;-\;3c_{1}\frac{\partial O_{\alpha z}}{\partial \alpha }\;+\;\frac{c_{1}}{R_{1}}\frac{\partial P_{\alpha z}}{\partial \alpha }+\;2c_{1}\frac{\partial^{2}P_{\alpha \beta }}{\partial \alpha \partial \beta }\;-f_{\mathrm{hygrothermal}}=\;-I_{0}\;\frac{\partial^{2}w_{0}}{\partial t^{2}}\;\\ -I_{1}\;\frac{\partial^{2}w_{1}}{\partial t^{2}}\;-\;I_{2}\frac{\partial^{2}w_{2}}{\partial t^{2}}\;+c_{1}I_{3}\;\frac{\partial^{3}u_{0}}{\partial \alpha \partial t^{2}}+c_{1}I_{3}\frac{\partial^{3}v_{0}}{\partial \beta \partial t^{2}}\;+\;c_{1}I_{4}\frac{\partial^{3}\phi_{\alpha }}{\partial \alpha \partial t^{2}}+c_{1}I_{4}\frac{\partial^{3}\phi_{\beta }}{\partial \beta \partial t^{2}}\\ -\frac{1}{2}\;c_{1}I_{5}\;\frac{\partial^{4}w_{1}}{\partial \alpha^{2}\partial t^{2}}-\frac{1}{2}\;c_{1}I_{5}\;\frac{\partial^{4}w_{1}}{\partial \beta^{2}\partial t^{2}}+\;c_{1}^{2}I_{6}\frac{\partial^{4}w_{0}}{\partial \alpha^{2}\partial t^{2}}\;+\;c_{1}^{2}I_{6}\frac{\partial^{4}w_{0}}{\partial \beta^{2}\partial t^{2}}\\ +\;c_{1}^{2}I_{6}\;\frac{\partial^{3}\phi_{\alpha }}{\partial \alpha \partial t^{2}}\;+\;c_{1}^{2}I_{6}\;\frac{\partial^{3}\phi_{\beta }}{\partial \beta \partial t^{2}}+c_{1}^{2}c_{3}I_{6}\frac{\partial^{4}w_{2}}{\partial \alpha^{2}\partial t^{2}}+c_{1}^{2}c_{3}I_{6}\frac{\partial^{4}w_{2}}{\partial \beta^{2}\partial t^{2}} \end{array}$$

(A8) $$\begin{array}{l} -\;\frac{\partial M_{\alpha \alpha }}{\partial \alpha }\;-\;c_{1}\frac{\partial P_{\alpha \alpha }}{\partial \alpha }+\;N_{\alpha z}\;-\frac{M_{\alpha z}}{R_{1}}\;+\;3c_{1}O_{\alpha z}-\frac{c_{1}}{R_{1}}\;P_{\alpha z}\;-\frac{\partial M_{\alpha \beta }}{\partial \beta }-c_{1}\frac{\partial P_{\alpha \beta }}{\partial \beta }\;=-I_{1}\frac{\partial^{2}u_{0}}{\partial t^{2}}-I_{2}\frac{\partial^{2}\phi_{\alpha }}{\partial t^{2}}\\ -c_{1}I_{3}\frac{\partial^{2}u_{0}}{\partial t^{2}}+\frac{1}{2}I_{3}\frac{\partial^{3}w_{1}}{\partial \alpha \partial t^{2}}-c_{1}I_{4}\frac{\partial^{3}w_{0}}{\partial \alpha \partial t^{2}}-2c_{1}I_{4}\frac{\partial^{2}\phi_{\alpha }}{\partial t^{2}}-c_{1}c_{3}I_{4}\frac{\partial^{3}w_{2}}{\partial \alpha \partial t^{2}}+\frac{1}{2}c_{1}I_{5}\frac{\partial^{3}w_{1}}{\partial \alpha \partial t^{2}}-c_{1}^{2}I_{6}\frac{\partial^{3}w_{0}}{\partial \alpha \partial t^{2}}\\ -c_{1}^{2}I_{6}\frac{\partial^{2}\phi_{\alpha }}{\partial t^{2}}-c_{1}^{2}c_{3}I_{6}\frac{\partial^{3}w_{2}}{\partial \alpha \partial t^{2}} \end{array}$$

(A9) $$\begin{array}{l} -\frac{\partial M_{\beta \beta }}{\partial \beta }\;-c_{1}\frac{\partial P_{\beta \beta }}{\partial \beta }\;+\;N_{\beta z}\;-\frac{M_{\beta z}}{R_{2}}\;+3c_{1}O_{\beta z}-\;\frac{c_{1}}{R_{2}}P_{\beta z}\;-\;\frac{\partial M_{\alpha \beta }}{\partial \alpha }-c_{1}\frac{\partial P_{\alpha \beta }}{\partial \alpha }\;=-I_{1}\frac{\partial^{2}v_{0}}{\partial t^{2}}-I_{2}\frac{\partial^{2}\phi_{\beta }}{\partial t^{2}}\\ -c_{1}I_{3}\frac{\partial^{2}v_{0}}{\partial t^{2}}+\frac{1}{2}I_{3}\frac{\partial^{3}w_{1}}{\partial \beta \partial t^{2}}-c_{1}I_{4}\frac{\partial^{3}w_{0}}{\partial \beta \partial t^{2}}-2c_{1}I_{4}\frac{\partial^{2}\phi_{\beta }}{\partial t^{2}}-c_{1}c_{3}I_{4}\frac{\partial^{3}w_{2}}{\partial \beta \partial t^{2}}+\frac{1}{2}c_{1}I_{5}\frac{\partial^{3}w_{1}}{\partial \beta \partial t^{2}}-c_{1}^{2}I_{6}\frac{\partial^{3}w_{0}}{\partial \beta \partial t^{2}}\\ -c_{1}^{2}I_{6}\frac{\partial^{2}\phi_{\beta }}{\partial t^{2}}-c_{1}^{2}c_{3}I_{6}\frac{\partial^{3}w_{2}}{\partial \beta \partial t^{2}} \end{array}$$

(A10) $$\begin{array}{l} \frac{M_{\alpha \alpha }}{R_{1}}\;-\;\frac{1}{2}\frac{\partial^{2}O_{\alpha \alpha }}{\partial \alpha^{2}}\;+\;\frac{M_{\beta \beta }}{R_{2}}\;-\;\frac{1}{2}\frac{\partial^{2}O_{\beta \beta }}{\partial \beta^{2}}\;+\;N_{zz}-\frac{1}{2R_{2}}\;\frac{\partial O_{\beta z}}{\partial \beta }\;-\;\frac{1}{2R_{1}}\;\frac{\partial O_{\alpha z}}{\partial \alpha }\;-\;\frac{\partial^{2}O_{\alpha \beta }}{\partial \alpha \partial \beta }\;=\;-I_{1}\frac{\partial^{2}w_{0}}{\partial t^{2}}\\ -\;\frac{1}{2}\;I_{2}\frac{\partial^{3}u_{0}}{\partial \alpha \partial t^{2}}\;-\;\frac{1}{2}\;I_{2}\frac{\partial^{3}v_{0}}{\partial \beta \partial t^{2}}\;-I_{2}\frac{\partial^{2}w_{1}}{\partial t^{2}}\;-\;\frac{1}{2}I_{3}\frac{\partial^{3}\phi_{\alpha }}{\partial \alpha \partial t^{2}}\;-\;\frac{1}{2}I_{3}\frac{\partial^{3}\phi_{\beta }}{\partial \beta \partial t^{2}}\;-\;I_{3}\frac{\partial^{2}w_{2}}{\partial t^{2}}\;+\;\frac{1}{4}I_{4}\frac{\partial^{4}w_{1}}{\partial \alpha^{2}\partial t^{2}}\\ +\frac{1}{4}I_{4}\frac{\partial^{4}w_{1}}{\partial \beta^{2}\partial t^{2}}-\frac{1}{2}c_{1}I_{5}\frac{\partial^{4}w_{0}}{\partial \alpha^{2}\partial t^{2}}-\frac{1}{2}c_{1}I_{5}\frac{\partial^{4}w_{0}}{\partial \beta^{2}\partial t^{2}}-\frac{1}{2}c_{1}I_{5}\frac{\partial^{3}\phi_{\alpha }}{\partial \alpha \partial t^{2}}-\frac{1}{2}c_{1}I_{5}\frac{\partial^{3}\phi_{\beta }}{\partial \beta \partial t^{2}}-\frac{1}{2}c_{1}c_{3}I_{5}\frac{\partial^{4}w_{2}}{\partial \alpha^{2}\partial t^{2}}\\ -\frac{1}{2}c_{1}c_{3}I_{5}\frac{\partial^{4}w_{2}}{\partial \beta^{2}\partial t^{2}} \end{array}$$

(A11) $$\begin{array}{l} \frac{1}{R_{1}}O_{\alpha \alpha }+c_{1}c_{3}\frac{\partial^{2}P_{\alpha \alpha }}{\partial \alpha^{2}}+\frac{1}{R_{2}}O_{\beta \beta }+c_{1}c_{3}\frac{\partial^{2}P_{\beta \beta }}{\partial \beta^{2}}+2M_{zz}+\frac{c_{1}c_{3}}{R_{2}}\frac{\partial P_{\beta z}}{\partial \beta }+\frac{c_{1}c_{3}}{R_{1}}\frac{\partial P_{\alpha z}}{\partial \alpha }+2c_{1}c_{3}\frac{\partial^{2}P_{\alpha \beta }}{\partial \alpha \partial \beta }\\ =\;-I_{2}\;\frac{\partial^{2}w_{0}}{\partial t^{2}}\;+\;c_{1}c_{3}I_{3}\frac{\partial^{3}u_{0}}{\partial \alpha \partial t^{2}}\;+\;c_{1}c_{3}I_{3}\;\frac{\partial^{3}v_{0}}{\partial \beta \partial t^{2}}\;-\;I_{3}\;\frac{\partial^{2}w_{1}}{\partial t^{2}}\;+\;c_{1}c_{3}I_{4}\;\frac{\partial^{3}\phi_{\alpha }}{\partial \alpha \partial t^{2}}\;+\;c_{1}c_{3}I_{4}\frac{\partial^{3}\phi_{\beta }}{\partial \beta \partial t^{2}}\\ -\;I_{4}\;\frac{\partial^{2}w_{2}}{\partial t^{2}}\;-\;\frac{1}{2}\;c_{1}c_{3}I_{5}\;\frac{\partial^{4}w_{1}}{\partial \alpha^{2}\partial t^{2}}\;-\;\frac{1}{2}c_{1}c_{3}I_{5}\;\frac{\partial^{4}w_{1}}{\partial \beta^{2}\partial t^{2}}\;+\;c_{1}^{2}c_{3}I_{6}\frac{\partial^{4}w_{0}}{\partial \alpha^{2}\partial t^{2}}\;+\;c_{1}^{2}c_{3}I_{6}\;\frac{\partial^{4}w_{0}}{\partial \beta^{2}\partial t^{2}}\\ +c_{1}^{2}c_{3}I_{6}\frac{\partial^{3}\phi_{\alpha }}{\partial \alpha \partial t^{2}}+c_{1}^{2}c_{3}I_{6}\frac{\partial^{3}\phi_{\beta }}{\partial \beta \partial t^{2}}+c_{1}^{2}c_{3}^{2}I_{6}\frac{\partial^{4}w_{2}}{\partial \alpha^{2}\partial t^{2}}+c_{1}^{2}c_{3}^{2}I_{6}\frac{\partial^{4}w_{2}}{\partial \beta^{2}\partial t^{2}} \end{array}$$

## References

[1] Feng L., Liu Z. (2011). Graphene in biomedicine: Opportunities and challenges. Nanomedicine.

[2] Delcea M., Möhwald H., Skirtach A.G. (2011). Stimuli-responsive lbl capsules and nanoshells for drug delivery. Adv. Drug Deliv. Rev..

[3] Cabrera C.R., Miranda F. (2014). Advanced Nanomaterials for Aerospace Applications.

[4] Du B.G., Zhang J.X., Zhu H.T., Zhou L.S. (2005). The application of nano-material in automobile. Commun. Stand..

[5] Tiginyanu I., Braniste T., Smazna D., Deng M., Schütt F., Schuchardt A., Stevens-Kalceff M.A., Raevschi S., Schürmann U., Kienle L. (2018). Self-organized and self-propelled aero-gan with dual hydrophilic-hydrophobic behavior. Nano Energy.

[6] Abbasi A., Park K., Bose A., Bothun G.D. (2017). Near-infrared responsive gold–layersome nanoshells. Langmuir.

[7] Villaverde A. (2011). Nanoparticles in Translational Science and Medicine.

[8] Timoshenko S.P., Woinowsky-Krieger S. (1959). Theory of Plates and Shells.

[9] Vlasov V.Z. (1964). General Theory of Shells And Its Application in Engineering.

[10] Niordson F.I. (2012). Shell Theory.

[11] Ciarlet P.G. (2000). Theory of Shells.

[12] Tornabene F., Fantuzzi N. (2014). Mechanics of Laminated Composite Doubly-Curvel Shell Structures: The Generalized Differential Quadrature Method and The Strong Formulation Finite Element Method.

[13] Fuller C., Fahy F.J. (1982). Characteristics of wave propagation and energy distributions in cylindrical elastic shells filled with fluid. J. Sound Vib..

[14] Mead D. (1996). Wave propagation in continuous periodic structures: Research contributions from southampton, 1964–1995. J. Sound Vib..

[15] Yuan F., Hsieh C. (1998). Three-dimensional wave propagation in composite cylindrical shells. Compos. Struct..

[16] Solaroli G., Gu Z., Baz A., Ruzzene M. (2003). Wave propagation in periodic stiffened shells: Spectral finite element modeling and experiments. Modal Anal..

[17] Wang X., Lu G., Guillow S. (2002). Stress wave propagation in orthotropic laminated thick-walled spherical shells. Int. J. Solids Struct..

[18] Golbahar Haghighi M., Malekzadeh P., Rahideh H., Vaghefi M. (2012). Inverse transient heat conduction problems of a multilayered functionally graded cylinder. Numer. Heat Transf. Part A Appl..

[19] Graff K.F. (2012). Wave Motion in Elastic Solids.

[20] Thai H.-T., Kim S.-E. (2015). A review of theories for the modeling and analysis of functionally graded plates and shells. Compos. Struct..

[21] Alijani F., Amabili M. (2014). Non-linear vibrations of shells: A literature review from 2003 to 2013. Int. J. Non-Linear Mech..

[22] Tornabene F., Fantuzzi N., Viola E., Batra R.C. (2015). Stress and strain recovery for functionally graded free-form and doubly-curved sandwich shells using higher-order equivalent single layer theory. Compos. Struct..

[23] Reddy J.N. (2004). Mechanics of Laminated Composite Plates and Shells: Theory and Analysis.

[24] Shimpi R., Patel H. (2006). A two variable refined plate theory for orthotropic plate analysis. Int. J. Solids Struct..

[25] Thai H.-T., Vo T.P., Nguyen T.-K., Kim S.-E. (2017). A review of continuum mechanics models for size-dependent analysis of beams and plates. Compos. Struct..

[26] Reddy J., Phan N. (1985). Stability and vibration of isotropic, orthotropic and laminated plates according to a higher-order shear deformation theory. J. Sound Vib..

[27] Amabili M. (2015). Non-linearities in rotation and thickness deformation in a new third-order thickness deformation theory for static and dynamic analysis of isotropic and laminated doubly curved shells. Int. J. Non-Linear Mech..

[28] Amabili M. (2015). A new third-order shear deformation theory with non-linearities in shear for static and dynamic analysis of laminated doubly curved shells. Compos. Struct..

[29] Carrera E., Brischetto S., Cinefra M., Soave M. (2011). Effects of thickness stretching in functionally graded plates and shells. Compos. Part B Eng..

[30] Neves A., Ferreira A., Carrera E., Cinefra M., Roque C., Jorge R., Soares C.M. (2013). Static, free vibration and buckling analysis of isotropic and sandwich functionally graded plates using a quasi-3d higher-order shear deformation theory and a meshless technique. Compos. Part B Eng..

[31] Thai H.-T., Kim S.-E. (2013). A simple quasi-3d sinusoidal shear deformation theory for functionally graded plates. Compos. Struct..

[32] She G.-L., Yuan F.-G., Karami B., Ren Y.-R., Xiao W.-S. (2019). On nonlinear bending behavior of fg porous curved nanotubes. Int. J. Eng. Sci..

[33] She G.-L., Yuan F.-G., Ren Y.-R., Liu H.-B., Xiao W.-S. (2018). Nonlinear bending and vibration analysis of functionally graded porous tubes via a nonlocal strain gradient theory. Compos. Struct..

[34] Karami B., Shahsavari D., Janghorban M. (2018). Wave propagation analysis in functionally graded (fg) nanoplates under in-plane magnetic field based on nonlocal strain gradient theory and four variable refined plate theory. Mech. Adv. Mater. Struct..

[35] Shahsavari D., Karami B., Li L. (2018). A high-order gradient model for wave propagation analysis of porous fg nanoplates. Steel Compos. Struct..

[36] Karami B., Shahsavari D., Li L., Karami M., Janghorban M. (2018). Thermal buckling of embedded sandwich piezoelectric nanoplates with functionally graded core by a nonlocal second-order shear deformation theory. Proc. Inst. Mech. Eng. Part C J. Mech. Eng. Sci..

[37] Heshmati M., Amini Y. (2019). A comprehensive study on the functionally graded piezoelectric energy harvesting from vibrations of a graded beam under travelling multi-oscillators. Appl. Math. Model..

[38] Karami B., Shahsavari D., Janghorban M. (2018). A comprehensive analytical study on functionally graded carbon nanotube-reinforced composite plates. Aerosp. Sci. Technol..

[39] Yee D.W., Schulz M.D., Grubbs R.H., Greer J.R. (2017). Functionalized 3d architected materials via thiol-michael addition and two-photon lithography. Adv. Mater..

[40] Liontas R., Jafary-Zadeh M., Zeng Q., Zhang Y.-W., Mao W.L., Greer J.R. (2016). Substantial tensile ductility in sputtered zr-ni-al nano-sized metallic glass. Acta Mater..

[41] Shahsavari D., Shahsavari M., Li L., Karami B. (2018). A novel quasi-3d hyperbolic theory for free vibration of fg plates with porosities resting on winkler/pasternak/kerr foundation. Aerosp. Sci. Technol..

[42] Yahia S.A., Atmane H.A., Houari M.S.A., Tounsi A. (2015). Wave propagation in functionally graded plates with porosities using various higher-order shear deformation plate theories. Struct. Eng. Mech..

[43] Lellep J., Majak J. (2000). Nonlinear constitutive behavior of orthotropic materials. Mech. Compos. Mater..

[44] Karami B., Janghorban M., Tounsi A. (2018). Variational approach for wave dispersion in anisotropic doubly-curved nanoshells based on a new nonlocal strain gradient higher order shell theory. Thin-Walled Struct..

[45] Dai H., Wang X. (2005). Stress wave propagation in laminated piezoelectric spherical shells under thermal shock and electric excitation. Eur. J. Mech. A/Solids.

[46] Wattanasakulpong N., Ungbhakorn V. (2014). Linear and nonlinear vibration analysis of elastically restrained ends fgm beams with porosities. Aerosp. Sci. Technol..

[47] Magnucki K., Malinowski M., Kasprzak J. (2006). Bending and buckling of a rectangular porous plate. Steel Compos. Struct..

[48] Şimşek M., Aydın M. (2017). Size-dependent forced vibration of an imperfect functionally graded (fg) microplate with porosities subjected to a moving load using the modified couple stress theory. Compos. Struct..

[49] Shahsavari D., Karami B., Fahham H.R., Li L. (2018). On the shear buckling of porous nanoplates using a new size-dependent quasi-3d shear deformation theory. Acta Mech..

[50] Karami B., Janghorban M., Shahsavari D., Tounsi A. (2018). A size-dependent quasi-3d model for wave dispersion analysis of fg nanoplates. Steel Compos. Struct..

[51] Arash B., Ansari R. (2010). Evaluation of nonlocal parameter in the vibrations of single-walled carbon nanotubes with initial strain. Phys. E Low-Dimens. Syst. Nanostruct..

[52] Ghavanloo E., Fazelzadeh S.A. (2013). Radial vibration of free anisotropic nanoparticles based on nonlocal continuum mechanics. Nanotechnology.

[53] Giwa A.M., Aitken Z.H., Jafary-Zadeh M., Liaw P.K., Zhang Y.-W., Greer J.R. Temperature effect on Small-Scale Deformation of Individual on the phases of Al0.7CoCrFeNi High Entropy Alloy HEA. https://mse.utk.edu.

[54] Ni X., Papanikolaou S., Vajente G., Adhikari R.X., Greer J.R. (2017). Probing microplasticity in small-scale fcc crystals via dynamic mechanical analysis. Phys. Rev. Lett..

[55] Eringen A.C., Edelen D. (1972). On nonlocal elasticity. Int. J. Eng. Sci..

[56] Lam D.C., Yang F., Chong A., Wang J., Tong P. (2003). Experiments and theory in strain gradient elasticity. J. Mech. Phys. Solids.

[57] Aifantis E.C. (2003). Update on a class of gradient theories. Mech. Mater..

[58] Ebrahimi F., Barati M.R., Dabbagh A. (2016). A nonlocal strain gradient theory for wave propagation analysis in temperature-dependent inhomogeneous nanoplates. Int. J. Eng. Sci..

[59] Karami B., Janghorban M., Tounsi A. (2017). Effects of triaxial magnetic field on the anisotropic nanoplates. Steel Compos. Struct..

[60] Karami B., Janghorban M., Tounsi A. (2018). Nonlocal strain gradient 3d elasticity theory for anisotropic spherical nanoparticles. Steel Compos. Struct..

[61] Khaniki H.B., Hosseini-Hashemi S. (2017). Buckling analysis of tapered nanobeams using nonlocal strain gradient theory and a generalized differential quadrature method. Mater. Res. Express.

[62] Nami M.R., Janghorban M. (2014). Resonance behavior of fg rectangular micro/nano plate based on nonlocal elasticity theory and strain gradient theory with one gradient constant. Compos. Struct..

[63] She G.-L., Yan K.-M., Zhang Y.-L., Liu H.-B., Ren Y.-R. (2018). Wave propagation of functionally graded porous nanobeams based on non-local strain gradient theory. Eur. Phys. J. Plus.

[64] She G.-L., Ren Y.-R., Yuan F.-G., Xiao W.-S. (2018). On vibrations of porous nanotubes. Int. J. Eng. Sci..

[65] Karami B., Janghorban M., Tounsi A. (2018). Galerkin’s approach for buckling analysis of functionally graded anisotropic nanoplates/different boundary conditions. Eng. Comput..

[66] Shahsavari D., Karami B., Mansouri S. (2018). Shear buckling of single layer graphene sheets in hygrothermal environment resting on elastic foundation based on different nonlocal strain gradient theories. Eur. J. Mech. A/Solids.

[67] Li L., Hu Y., Ling L. (2016). Wave propagation in viscoelastic single-walled carbon nanotubes with surface effect under magnetic field based on nonlocal strain gradient theory. Phys. E Low-Dimens. Syst. Nanostruct..

[68] Xiao W., Li L., Wang M. (2017). Propagation of in-plane wave in viscoelastic monolayer graphene via nonlocal strain gradient theory. Appl. Phys. A.

[69] Karami B., Shahsavari D., Janghorban M., Li L. (2018). Wave dispersion of mounted graphene with initial stress. Thin-Walled Struct..

[70] Mehralian F., Beni Y.T., Zeverdejani M.K. (2017). Nonlocal strain gradient theory calibration using molecular dynamics simulation based on small scale vibration of nanotubes. Phys. B Condens. Matter.

[71] Karami B., Shahsavari D., Li L. (2018). Temperature-dependent flexural wave propagation in nanoplate-type porous heterogenous material subjected to in-plane magnetic field. J. Therm. Stresses.

[72] Barati M.R. (2017). On wave propagation in nanoporous materials. Int. J. Eng. Sci..

[73] Karami B., Shahsavari D., Li L. (2018). Hygrothermal wave propagation in viscoelastic graphene under in-plane magnetic field based on nonlocal strain gradient theory. Phys. E Low-Dimens. Syst. Nanostruct..

[74] Shahsavari D., Karami B., Li L. (2018). Damped vibration of a graphene sheet using a higher-order nonlocal strain-gradient kirchhoff plate model. Comptes Rendus Mécanique.

[75] She G.-L., Yuan F.-G., Ren Y.-R. (2018). On wave propagation of porous nanotubes. Int. J. Eng. Sci..

[76] Karami B., Janghorban M., Li L. (2018). On guided wave propagation in fully clamped porous functionally graded nanoplates. Acta Astronaut..

[77] Karami B., Shahsavari D., Nazemosadat S.M.R., Li L., Ebrahimi A. (2018). Thermal buckling of smart porous functionally graded nanobeam rested on kerr foundation. Steel Compos. Struct..

[78] Askes H., Aifantis E.C. (2009). Gradient elasticity and flexural wave dispersion in carbon nanotubes. Phys. Rev. B.

[79] Lim C., Zhang G., Reddy J. (2015). A higher-order nonlocal elasticity and strain gradient theory and its applications in wave propagation. J. Mech. Phys. Solids.

[80] Chen H., Wang A., Hao Y., Zhang W. (2017). Free vibration of fgm sandwich doubly-curved shallow shell based on a new shear deformation theory with stretching effects. Compos. Struct..

[81] Amabili M. (2018). Nonlinear Mechanics of Shells and Plates in Composite, Soft and Biological Materials.

[82] Ebrahimi F., Barati M.R. (2016). Wave propagation analysis of quasi-3d fg nanobeams in thermal environment based on nonlocal strain gradient theory. Appl. Phys. A.

[83] Shahsavari D., Karami B., Janghorban M., Li L. (2017). Dynamic characteristics of viscoelastic nanoplates under moving load embedded within visco-pasternak substrate and hygrothermal environment. Mater. Res. Express.

[84] Karami B., Shahsavari D., Karami M., Li L. (2018). Hygrothermal wave characteristic of nanobeam-type inhomogeneous materials with porosity under magnetic field. Proc. Inst. Mech. Eng. Part C J. Mech. Eng. Sci..

[85] Pandey A., Patel A.K., Kumar V., Sharma R.K., Kanhed S., Nigam V.K., Keshri A., Agarwal A., Balani K. (2018). Enhanced tribological and bacterial resistance of carbon nanotube with ceria-and silver-incorporated hydroxyapatite biocoating. Nanomaterials.

[86] Pei Y., Zhong H., Wang M., Zhang P., Zhao Y. (2018). Effect of contact pressure on the performance of carbon nanotube arrays thermal interface material. Nanomaterials.

[87] Pan J., Liu S., Yang Y., Lu J. (2018). A highly sensitive resistive pressure sensor based on a carbon nanotube-liquid crystal-pdms composite. Nanomaterials.

[88] Song M., Yang J., Kitipornchai S. (2018). Bending and buckling analyses of functionally graded polymer composite plates reinforced with graphene nanoplatelets. Compos. Part B Eng..

[89] Huang J., Her S.-C., Yang X., Zhi M. (2018). Synthesis and characterization of multi-walled carbon nanotube/graphene nanoplatelet hybrid film for flexible strain sensors. Nanomaterials.

[90] Slattery A., Shearer C., Shapter J., Blanch A., Quinton J., Gibson C. (2018). Improved application of carbon nanotube atomic force microscopy probes using peakforce tapping mode. Nanomaterials.

[91] Giwa A.M., Liaw P.K., Dahmen K.A., Greer J.R. (2016). Microstructure and small-scale size effects in plasticity of individual phases of al 0.7 cocrfeni high entropy alloy. Extreme Mech. Lett..

[92] Rosenkrantz E., Bottero A., Komatitsch D., Monteiller V. (2019). A flexible numerical approach for non-destructive ultrasonic testing based on a time-domain spectral-element method: Ultrasonic modeling of lamb waves in immersed defective structures and of bulk waves in damaged anisotropic materials. NDT E Int..

[93] Wu J., Li X., Cao W. (2013). Flexural waves in multi-walled carbon nanotubes using gradient elasticity beam theory. Comput. Mater. Sci..

[94] Papargyri-Beskou S., Polyzos D., Beskos D. (2009). Wave dispersion in gradient elastic solids and structures: A unified treatment. Int. J. Solids Struct..

[95] Li L., Hu Y., Ling L. (2015). Flexural wave propagation in small-scaled functionally graded beams via a nonlocal strain gradient theory. Compos. Struct..

